# A New Method for Estimating the Clamping Force of Shrink Sleeve Labels

**DOI:** 10.3390/ma11122544

**Published:** 2018-12-14

**Authors:** Jarosław Szusta, Adam Tomczyk, Özler Karakaş

**Affiliations:** 1 Faculty of Mechanical Engineering, Department of Mechanics and Applied Computer Science, Bialystok University of Technology, Wiejska 45C Str., 15-351 Białystok, Poland; j.szusta@pb.edu.pl (J.S.); a.tomczyk@pb.edu.pl (A.T.); 2 Masterpress S.A., Kuronia 4 Str, 15-569 Białystok, Poland; 3 Mechanical Engineering Department, Engineering Faculty, Pamukkale University, 20070 Kinikli, Denizli, Turkey

**Keywords:** shrink sleeve, thin film, principal strains, strain gauge, thin-walled container

## Abstract

The paper presents an original method for estimating the shrink sleeve label compressive force on packaging. One of the most popular methods of measuring deformations was used, i.e., the electrical resistance strain gauge measurement. It was assumed that the packaging was a thin-walled axially symmetrical vessel. The packing walls on one side are loaded with internal pressure generated by heating the liquid contained inside the packaging. On the other side, the film shrinking on the packaging generates additional deformation. By measuring the changes in circumferential deformations in the shrinking process at various packaging heights, it is possible to infer the uniformity of the film compressive force. Results of research on changes of these deformations over time with different intensity values of the shrinkage medium were presented.

## 1. Introduction

**Figure 1:**
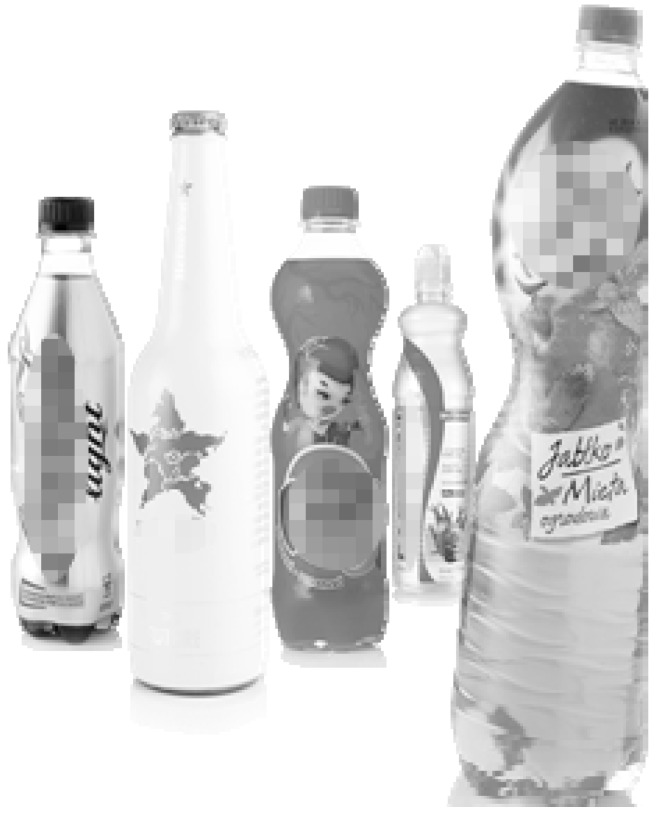
Sample packaging with heat-shrink film labels. (Adapted from resources of Masterpress S.A.).

Heat shrink films are now widely used in various industries to produce all kinds of labels used on glass, metal and plastic packaging (Figure 1). The labels on bottles that are pleasing to the eye, making the item more attractive and appealing to the customer in the shops. This often determines the marketing success of the product and thus the manufacturer’s income.

Along with the relevant developments in the production technology of heat-shrinkable sleeve labels, this method gained distinct advantages over other forms of labeling. Among the many essential advantages, we can distinguish the following:

- excellent visual effects;
- greater design options when compared to traditional printing methods;
- the possibility of covering the entire surface of containers even with complicated, unusual shapes, for which it would be difficult to adapt self-adhesive labels;
- saving costs compared to using many labels on the container;
- additional benefits resulting from the possibility of securing the entire container;
- the possibility of printing also on the inner part of the sleeve;
- ensuring complete resistance to abrasion of prints by printing the inside of the sleeve;
- increasing the brands’ impact through excellent presentation at sales points;
- ideal properties to adapt to marketing campaigns, such as so-called multipacks;
- possibility of making perforations, breakable parts, coupons, etc.;
- cost-effective low-volume printing in flexographic technology.

Based on these clear advantages, it is safe to predict a significant increase in the scale of production of shrink sleeve labels in the coming years. In fact, it would be reasonable to state that the labels of this type will dominate the packaging of products in the near future.

Obtaining the desired visual effect depends on a number of factors, such as the film shrinkage process parameters, the shape and size of the packaging or the selection of material with respect to chemical and strength properties. Knowledge of material characteristics in turn allows for the application of appropriate parameters of the film printing process and its subsequent shrinking on packaging. The selection of suitable strength parameters is primarily determined by the strength of the film compressive force, its corresponding deformation, lack of undesirable cracks etc.

Shrink sleeve films are of interest to many authors. This has been the case since the beginning of the first applications of this type of film in 1958, when it was possible to combine styrene with synthetic rubber [1,2].The literature provides a number of works on tests of various properties, including mechanical properties of the films. An extensive review of various films in terms of strength parameters can be found in work of [3]. Strength tests of polymers have also been described in studies by Avérous et al. [4], Matzinos et al. [5], Mo et al. [6], Srinivasa and Tharanathan [7], Tserki et al. [8,9], Wang et al. [10], Zhang et al. [11], Zhang et al. [12], Szusta et al. [13]. Many studies placed emphasis on one of the cheapest and most commonly available materials used for labels, starch-based polymers [4,10]. It should also be noted that the method of manufacturing polymer films has a significant effect on their mechanical properties. This particularly pertains to cross-linked polymers such as PE, PP, acrylate, where the cross-linking method has a decisive influence on the aforementioned properties [14,15]. Very good mechanical properties are obtained for oriented layered polymers [16]. The number of layers in materials of this type usually falls within the range of 2–7. Besides strength properties, much attention is also devoted to shaping optimal optical and visual properties [17,18].

The quality of the label shrinking process on the packaging is assessed by visual inspection of the outer container with the label applied at the present time. The adhesion of the label to the container walls is checked and the compressive force is assessed by the trial involving moving or rotating the label on the container. Such verification is not objective and does not give unambiguous results. There is a lack of more accurate methods to assess the uniformity of the compressive force of the label on the packaging. The main purpose of the manuscript is to present a new, original method, which is quantitative rather than just qualitative, in order to improve shrinkage process assessment. This method can be used to accurately estimate the film clamping force, and above all the uniformity of the force. In addition, using this method of preliminary assessment, the optimal parameters of the shrinking process such as temperature, the intensity of the working fluid stream or the distribution of the working fluid sources inside the shrink tunnel can be determined.

One of the essential elements affecting the energy intensity of the label’s shrinkage process is the shape and size of the packaging on which the heat shrink film is applied. It is reasonable that one should strive for a shape that will facilitate uniform distribution of the film compressive force on the packaging (e.g., a bottle) in the direction of the package axis i.e., in the vertical direction, and will have an appropriate value. The film can be properly adhered to the packaging walls by the appropriate selection of these two parameters, i.e., the value of the shrinkage force and uniformity of shrinkage in the vertical direction. This prevents deformation of the film itself as well as the packaging, for example due to too tight compression of the film. Deformations primarily result in various distortions of graphics that are already on the shrunk film. Consequently, incorrectly compressed film can make the final product visually unattractive to the prospective buyer.

## 2. Materials and Methods

### 2.1. Production Process of a Heat-Shrink Label

The production process of the heat-shrink label begins with the selection of the type of film on which the imprint will be applied. The most popular materials used for the production of the sleeve are: polyolefin, PP, PE, PVC, OPS and PET. PET heat-shrinkable film is ideal for general-purpose packaging due to its properties such as:

- low price and availability worldwide;
- high gloss and transparency;
- high strength and elasticity—the shape of the packaged products do not warp and provides good formability;
- high shrinkage at low temperatures in a wide range of shrinkage ratios;
- cold resistance, making it suitable for labeling products stored at low temperatures.

Heat-shrinkable films are usually printed using the flexographic printing technique with appropriate paints. According to general trends in flexographic printing, the most commonly used paints are curable paint fixations and, more often, radical-fixed paints. Their advantages, such as high gloss, elasticity, as well as resistance to low and high temperatures, make them particularly well suited for printing heat-shrinkable sleeves. The most important feature that determines the type of paint used is its ability to shrink with the substrate. The transverse shrinkage often required for atypical shapes can reach 70%.

**Figure 2:**
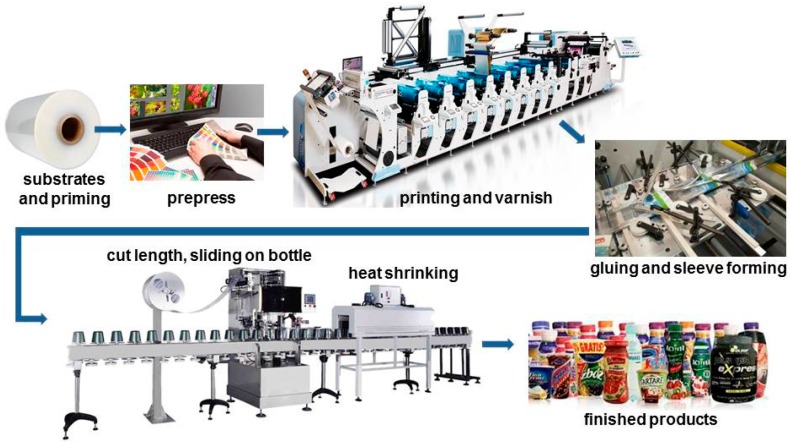
The production and application process of the heat-shrinkable label. (Adapted from resources of Masterpress S.A.).

The technological process of packaging production in shrink sleeve technology is multi-stage. First, the foil web is printed. In the next stage, after gluing the edges of the web, a sleeve is formed which is then wound up so that a role is created. Afterwards the sleeve is cut on the so-called “utilities”, i.e., individual labels, which are then manually or machine-applied to the packaging. The last procedure in the production process is shrink sleeve packaging process. This is usually done in a special tunnel where, using heated air or steam, the labels shrink the packaging thoroughly. Figure 2 shows the production and application process of the shrink-sleeve label on the packaging.

### 2.2. Measurement Method

#### 2.2.1. Axially Symmetrical Thin-Walled Vessels

**Figure 3:**
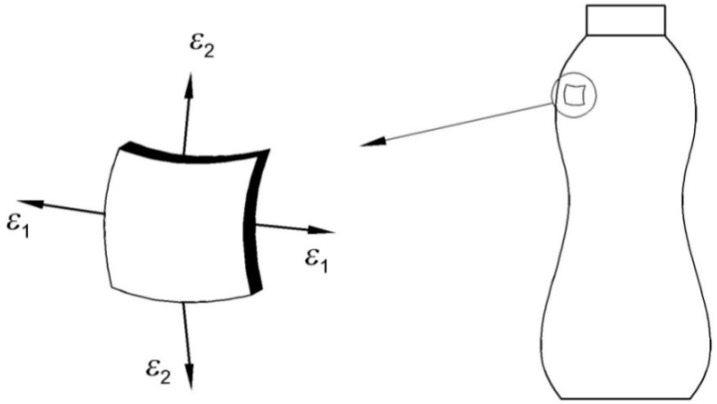
Schematic representation of principal strains in the walls of a sample axially symmetrical thin-walled vessel.

Most of the packaging on which the shrink sleeve labels are applied are axially symmetrical. It can be assumed that these are thin-walled axially symmetrical vessels loaded with internal pressure. This pressure is generated by the presence of fluid inside the container. It can additionally increase its volume due to the temperature increase inside the shrink tunnel. This results in the appearance of positive container wall deformations. At the same time, additional deformations are usually generated with the opposite sign as a result of the film compression. Therefore, we deal with an axially symmetrical thin-walled vessel loaded by pressure evenly distributed with respect to the axis of symmetry. In such cases, it can be assumed that the stress in the walls of the container corresponds to the plane stress case (Figure 3). The generalized Hooke’s laws expressed in terms of principal stresses have the following form (e.g., [19]):

(1) $$\sigma_{1}=\frac{E}{1-v^{2}}\left(\varepsilon_{1}+v\varepsilon_{2}\right),\;\sigma_{2}=\frac{E}{1-v^{2}}\left(\varepsilon_{2}+v\varepsilon_{1}\right)$$

where *ε*_1_, *ε*_2_—principal strains, *σ*_1_, *σ*_2_—principal stresses, *E*—Young’s modulus, *ν*—Poisson’s ratio.

When circumferential deformations *ε*_1_ are known, the compressive force of the film and the uniformity of the compression can be estimated.

With the stress value given by the provided Equation (1), it is possible to propose the determination of the circumferential clamping force as a force acting in a narrow band of the container. Assuming that this band is subjected to compression in the circumferential direction, this *F*_1_ force can be determined by multiplying the stresses *σ*_1_ by the cross-sectional area *S* of the band:

(2) $$F_{1}=\sigma_{1}S$$

However, it should be noted that, in fact this force is not the force of the film clamping, but the force generated in the wall of the packaging due to the clamping of the film. But of course, the nature of changes in this force is the same as the nature of changes in the clamping force of the film. On the other hand, the force in the film has relatively higher value.

#### 2.2.2. Electrical Resistance Strain Gauge Measurement

The most popular method of measuring deformations is the electrical resistance strain gauge measurement (e.g., [20]). If the values of the principal strain directions are not known, strain rosettes are usually used. However, in the analyzed case of the axially symmetrical vessels it can be assumed that these directions are known (Figure 3). Therefore, linear strain gauges were used. They were applied only along the circumferential direction 1. Direction 2 was omitted in measurements because the film shrinks only in the circumferential direction. In the vertical direction, the film shrinkage is negligible.

The probability of gluing the strain gauge ideally along the principal direction is low. However, this is not a problem, since the deviation of the strain gauge axis from this direction by up to several degrees will not significantly change the measurement results in the case concerned. Strain gauges were installed on the outer wall of the container at different heights along the main direction 1. This allowed us to estimate the strength of the film compressive force at different heights of the container, and to determine the possible non-uniformity of the compression.

By studying the deformations for various configurations of nozzles, e.g., steam nozzles and their various shapes, it is possible to determine the most optimal solution where the film compression at different levels will be similar. Testing using the presented method allowed for continuous recording and analysis of deformations (and hence stresses). Thus, changes could be estimated in the film compressive force from the moment of the steam blow until cooling.

#### 2.2.3. Test Stand

**Figure 4:**
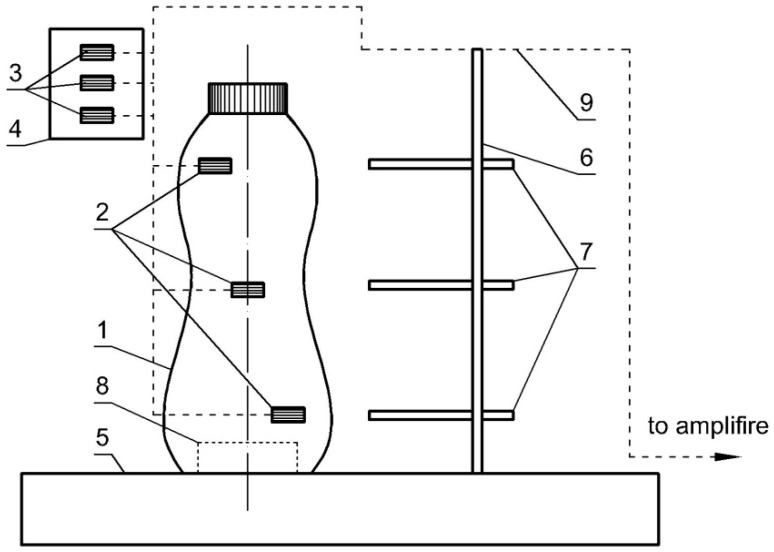
Schematic representation of a container with the attached strain gauges on a stand: 1—container, 2—strain gauges, 3—compensating strain gauges, 4—unloaded material, 5—base, 6—mandrel, 7—thermocouples, 8—magnet, 9—wiring.

The proposed method uses a reference container (1) (Figure 4), where strain gauges (2) are installed to analyze the deformations in the plastic parts. The number of strain gauges depends on the container height. The strain gauges were located in the height direction of the container along the helical line to cover the entire circumference of the tested packaging. Strain gauges (2) were installed along the first principal direction in the half-bridge system, i.e., each of the strain gauges had a compensator (3) that was glued onto a piece of unloaded material (4) identical to the container material. Such arrangement allows for the compensation of elevated temperature generated during the steam blow, e.g., steam in the tunnel. Additionally, after installing all strain gauges and necessary electrical wires, the gauges themselves and the exposed electrical connectors were protected with a special flexible polyurethane coating against the effects of moisture generated inside the tunnel.

The location of strain gauges must be sufficiently distant from places where the stress distributions in the container walls may be inhomogeneous. The areas in the immediate vicinity of the bottom of the container, its threaded outlet, as well as the minimal thickness of the wall resulting from the process of injecting the liquid material into the split mold should be avoided.

In order to ensure the stability of the container moving inside the heat tunnel with the gauges installed, and to allow the temperature to be recorded in the vicinity of each strain gauge, the container was mounted on a stand (Figure 4) consisting of the base (5), the mandrel (6) and the installed measuring thermocouples (7).

**Figure 5:**
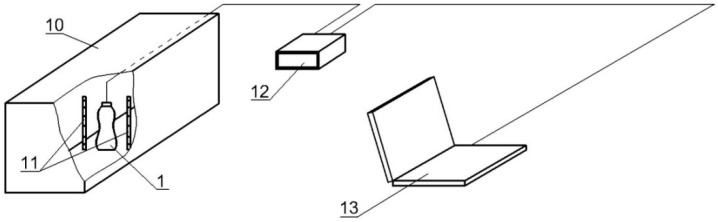
Schematic view of the measuring stand: 1—shrink wrapped film tested, 10—shrink tunnel, 11—nozzle layout fed from a steam generator, 12—measuring amplifier, 13—portable computer.

The base weight should provide a stable container position when moving in the shrink tunnel (10) (Figure 5) between the steam nozzle systems (11). The container (1) is mounted on the base of the stand by means of a magnet (8) (Figure 4) located inside its bottom. The wiring (9) is attached to the mandrel (6). This solution eliminates wiring movements between the stand pin and the container, and thus eliminates the impact of these movements on the deformation of the container walls. The signal from the strain gauges is transmitted to a special 8-channel HBM Quantum MX840A (Darmstadt, Germany) measuring amplifier (12) (Figure 5). Data is logged via a portable computer (13) with specialised software (Catman DAQ) for operating the amplifier.

The essence of the study is to set up a stand (including a container with strain gauges on which heat shrink film is applied) on a belt conveyor before entering the shrink tunnel and starting the conveyor and recording equipment. As the conveyor moves, the tooled container moves inside the tunnel, passing through heating sections. As a result of the appropriate sequence of heat impacts, the film compressed on the container exerts pressure on its walls. Film compression is recorded by strain gauges as circumferential deformations at various levels. During the testing and calibration of the test stand, several dozen tests were performed. However, the results of seven of them were presented in the manuscript.

It should be noted that the specific design and shape of the steam nozzles in the shrink tunnel is appropriate for the packaging of a particular shape and size. With the use of presented measurement method containers of different shapes and sizes can be analyzed. At the same time, testing the same packaging with different configurations of the nozzle system and different nozzle shapes will optimize this layout.

**Figure 6:**
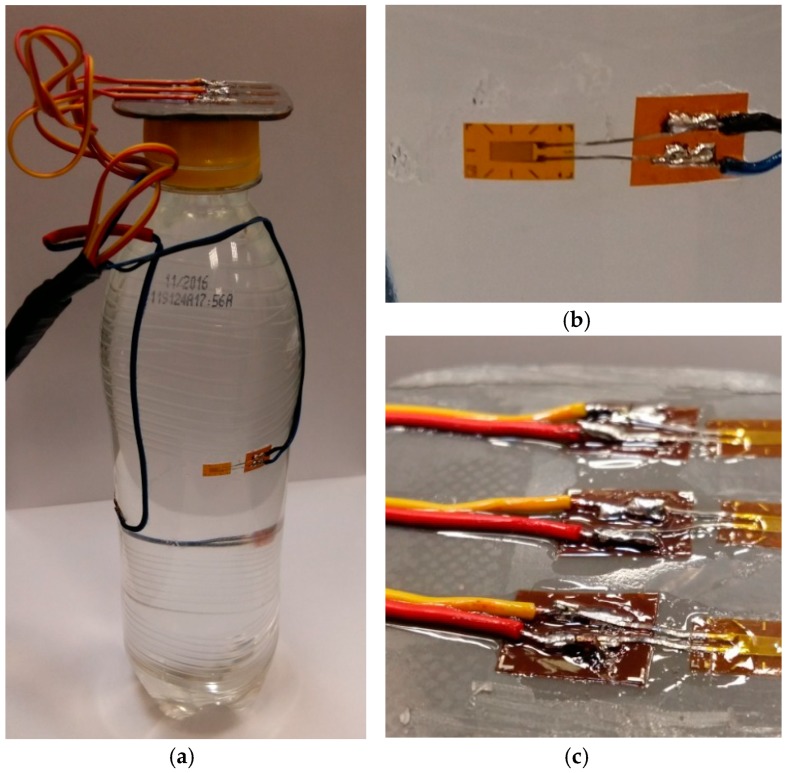
Configurations of the attached strain gauges on test container: (**a**) a bottle with strain gauges glued on three different heights; (**b**) a single strain gauge; (**c**) compensating strain gauges.

**Figure 7:**
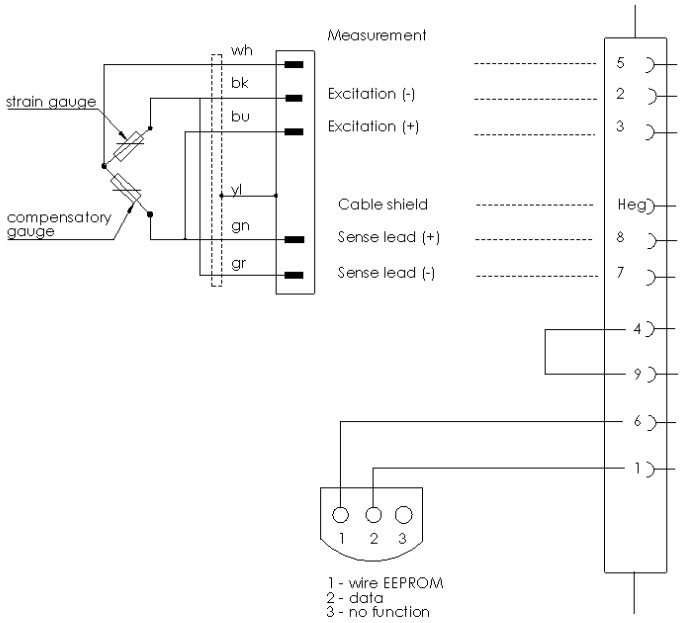
Diagram of connection of strain gauges in a half-bridge configuration.

The primary test piece was a PET bottle of 500 mL capacity with dedicated heat shrink film. Strain gauges were installed on the walls of the bottle. The tests were carried out with the use of special strain gauges 1-LY18-3/120A manufactured by HBM (Figure 6), intended for analyzing the deformations in plastic elements. Strain gauges were glued using special, elastic Z 70 adhesive manufactured by HBM. Exposed wiring harnesses were covered with two layers of PU 140 polyurethane protective coating to protect them from exposure to moisture. Strain gauges were installed in a half-bridge configuration, as shown in Figure 7.

It should be taken into consideration that the influence of connecting wires on the measurement results cannot be excluded. Determining the amount of the influence of these wiring would require measuring the strain with other methods, preferably non-contact, which in the conditions of the steam tunnel would be extremely difficult or can even be impossible. However, it is also important to note that all tests were carried out with the same wiring configuration. Thus, the possible error was always the same and negligible.

**Figure 8:**
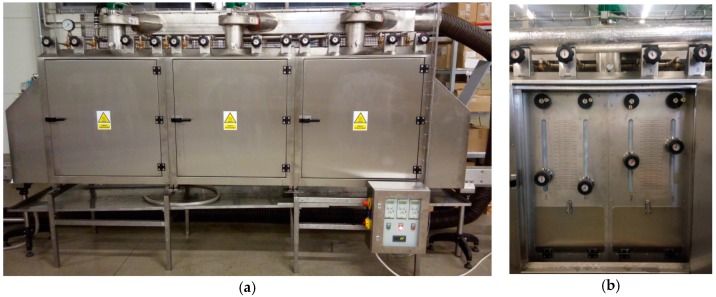
Photographs of: (**a**) the shrink tunnel in which the test was carried out; (**b**) the control panel of the steam flow rate in a sample section of the tunnel.

A single bottle is fitted with three strain gauges mounted at three different heights (Figure 4). Further in this paper these gauges are numbered, starting from the bottom of the bottle: the bottom gauge, the middle gauge and the top gauge. It is worth mentioning that, as in mass production, the bottle is filled with liquid (water) which prevents permanent deformation of the material in contact with hot steam. The study was carried out in a shrink tunnel (Figure 8a) equipped with three steam sections with each section composed of four systems of steam nozzles in an adjustable position for a total of twelve steam sections. Each of valves allowed independent control over the amount of steam generated and the position of each section from the control panel (Figure 8b).

This solution allows adjusting the steam flow to packages of varying heights and shapes. During the tests, the steam nozzle systems were arranged at equal distances along the length of the tunnel. However, the vertical position of the nozzles was adjusted to the shape of the tested object. It should be acknowledged that tunnels with different numbers of steam sections are used in industry.

The “0” value set on the valve of each section corresponds to the total steam cut off, while “10” indicates the maximum stream flow. The tests were carried out for various vapor flow rate settings in each section, however in line with the tunnel operator’s long-term experience, which means that the values set on the control panel were close to the values that had been used so far with good shrinkage quality. The values of settings used for the tests are shown in Table 1. Cut sleeve film with a diameter of 68 mm was used for testing.

## 3. Results

**Figure 9:**
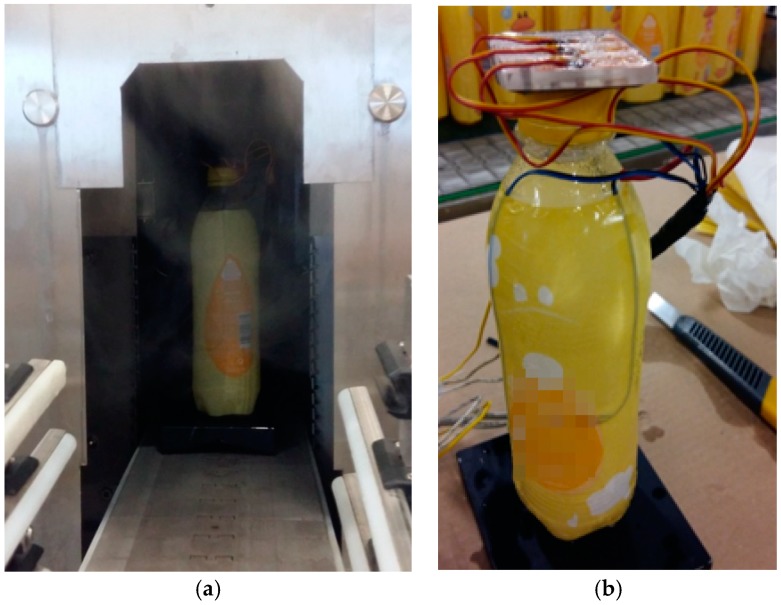
The stand with the bottle and shrunk film: (**a**) at the steam tunnel outlet; (**b**) after cooling.

The tests consisted in continuous recording of deformation changes in three strain gauges affixed to the container walls while the container was moving inside the shrink tunnel. At the same time, it should be noted that the maximum ambient temperature of the gauges during the tests (during the steam blow) recorded by thermocouples varied by 2–3 °C for subsequent repetitions and was in the range of 70–90 °C. Therefore, no additional results of this temperature analysis are presented in this report. It is worth emphasizing that after leaving the steam tunnel, the container was cooled to a temperature of approx. 30–31 °C. During the cooling stage, the compressed film was removed from the container with great caution so as not to damage the strain gauges. The view of the shrink-wrapped container at the tunnel outlet and after cooling is shown in Figure 9.

**Figure 10:**
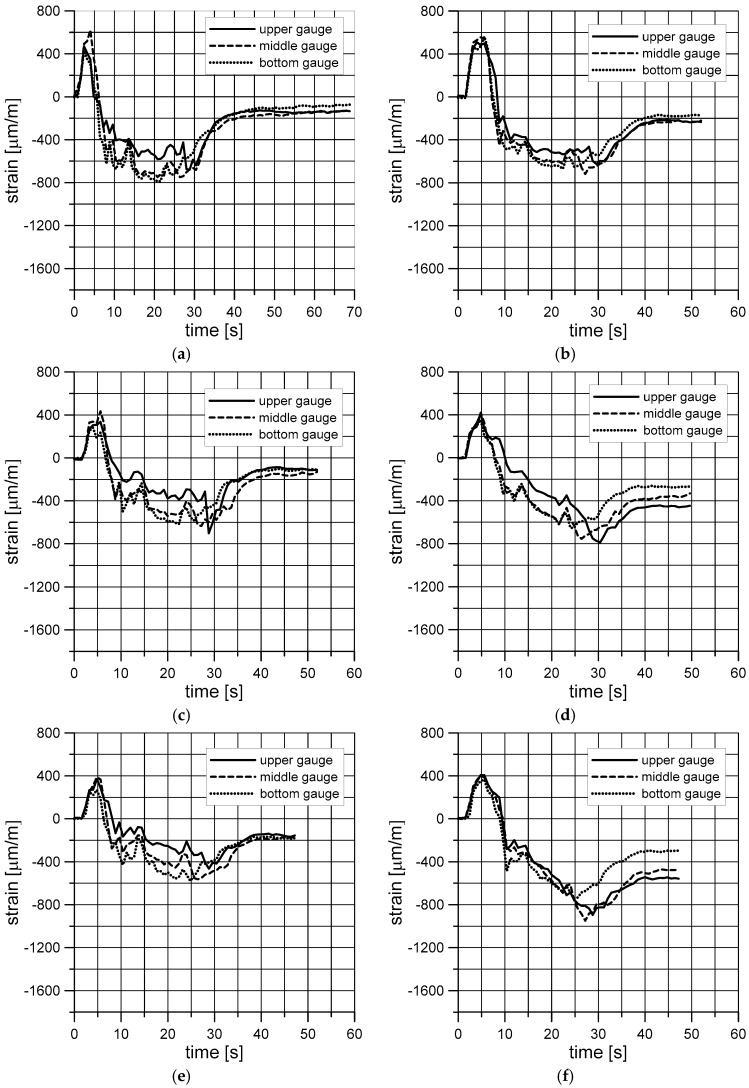

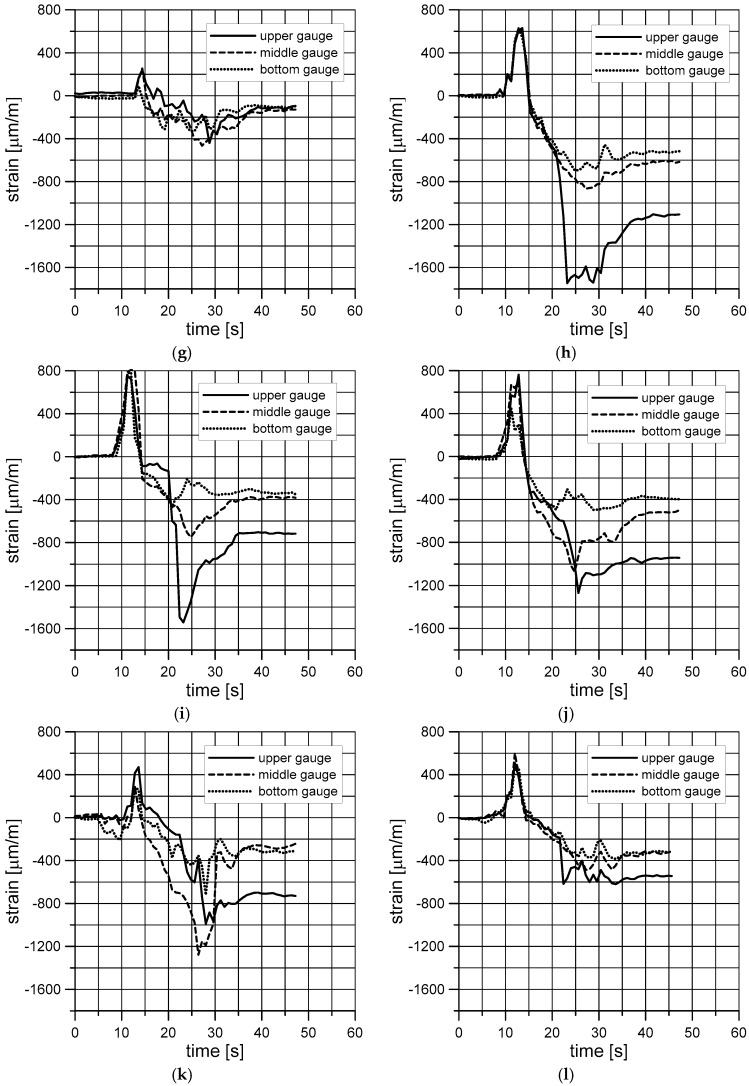

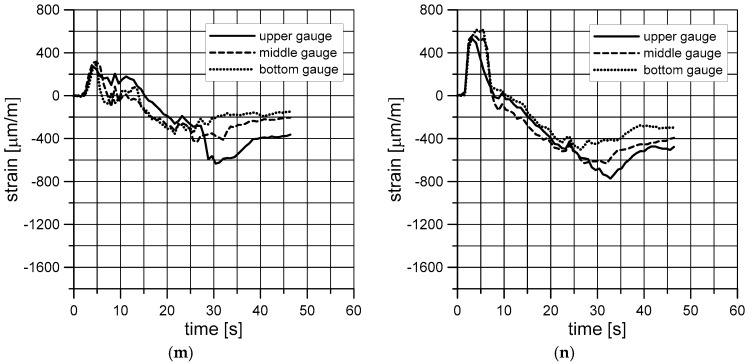
Averaged results obtained for different valve settings controlling the individual steam sections: (**a**,**b**) series 1; (**c**,**d**) series 2; (**e**,**f**) series 3; (**g**,**h**) series 4; (**i**,**j**) series 5; (**k**,**l**) series 6; (**m**,**n**) series 7.

The first few tests were intended to check the entire measuring system for correct function and made it possible to obtain an overview of the function of the individual shrink sections of the tunnel. It should be emphasized that due to the violent impact of steam at elevated temperatures, water contained in a sealed container increased its volume thus causing an increase in the pressure inside the container. This entailed an immediate strain gauge response, which is clearly indicated by a sharp increase in deformations. At the same time, due to the increased temperature, the material of the container (PET) shrank, which was also recorded by strain gauges. In order to isolate the effects of increased pressure inside the container and the container material heat shrinkage from the effects of film compression, a series of measurements was carried out for the container with no film and with the shrinking film on it. Each time the container was cooled down to a temperature of approximately 30–31 °C, which prevented the continuous heating of water in the bottle in subsequent measurements and thus the repeatability of the measurements. Each series consisted of six measurements: three for a container with no film and three for a container with shrink film on it. The results of these tests were averaged with respect to the strain gauges and are shown in Figure 10. The charts shown in Figure 10a,c,e,g,i,k,m refer to subsequent measurements of deformation of the container walls with no compressing film. The other charts, i.e., Figure 10b,d,f,h,j,l,n pertain to measurements with the film on.

## 4. Discussion

The results of all series of tests show that the steam impact on the container passing through the first active section caused a sharp increase in deformation at all three levels analyzed. In most cases, the maximum deformation level at three different heights is similar. However, the level of these deformations is different for different series. A sharp increase in deformation is due to an increase in the pressure inside the container as a result of water being heated inside the container. After the increase, a sharp decrease in deformation occurs. This should be explained by the heat shrinkage of the material of which the container (PET) was made. However, this shrinkage takes place a moment after the pressure increase due to the heating of water inside the container and is clearly dependent on the valve settings of the individual sections. Any further decrease in deformation is much more gradual due to the persistence of the elevated temperature at a relatively constant level. Yet, the oscillations of the deformation waveforms over time can be seen clearly as the container is passing through subsequent steam sections. This involves a slight decrease in temperature in the areas between the steam nozzle outlets of the individual sections. This is particularly evident in Figure 10a,c,e,g where each of the peaks corresponds to the vapor impact in each section. Eventually, in each case strain gauges always confirmed the presence of compression in the container walls. The degree of this compression depended on such factors as the steam flow rate.

For identical settings of all steam sections (Figure 10a–f), a steam blow is clearly marked at the height of each section. Such clear regularity cannot be observed in the other figures. Increasing the settings from 2.5 to 5 and 7 caused a decrease in the maximum value at the first rapid load variation. It can be explained by the fact that a higher temperature caused a faster container material response, which compensated for the faster increase in pressure due to water being heated. At the same time, for the same first three series, the value of the compressive deformation at the end of the measurement increased as the settings increased, both for the container with and without the shrink film applied. This is mainly due to higher shrinkage of the container material at higher temperatures. However, the effect of film compression is clearly noticeable here. Compression values for a container with the film applied are always higher than for film-free containers. The biggest differences are always for the upper gauge, while the smallest ones are for the bottom gauge. An increase in the settings caused higher divergence between the gauges located at three different heights.

The charts shown in Figure 10g–j correspond to the situation of shortening the steam tunnel by four sections (Figure 10g,h) and by eight sections (Figure 10i,j), while maintaining a low steam flow rate. For only four middle sections in operation, there was a sharp increase in deformation at three levels to a value of approximately 800 μm/m for a film-free container. For a container with the film applied, this increase was significantly lower due to the compressing effect of the film. In these cases, despite the low steam flow rate, the highest compressive force of the film was obtained on the packaging, especially at its top.

The last four drawings (Figure 10k–n) show the effects of a gradual increase in the shrinking medium flow rate in subsequent sections (Figure 10k,l) and its gradual decrease (Figure 10m,n). In the first case after the standard first spike, we can observe another sharp spike in the direction of the compressive strain, which should be attributed to a strong steam stroke in the last sections. In the latter case, the first rapid increase in deformation caused by water being heated inside the container was limited by equally rapid heat shrinkage of the material. It is worth noting that the high deformation value persisted for a few seconds here (Figure 10m,n), as opposed to the former (Figure 10c).

Due to the novelty of the method, currently it is difficult to provide typical statistical processing of experimental data such as the average of values and standard deviation alongside Figure 10 as the results are too little so far. However, in the future, the authors intend to do more repetitions of each test and present comprehensive results of statistical analysis.

## 5. Conclusions

To conclude, it should be stated that the highest film compression values were obtained for the cases of the “shortened” steam tunnel. In this case, however, the divergence between the compressive forces at three different levels was highest. The lowest divergence was obtained for identical settings in all steam sections. Divergences in these values are lowered as the shrinking medium flow rate stream decreased. However, the film compression was the smallest in these cases.

What plays a significant role in the film shrinking process is the liquid filling the packaging. As a result of a sudden steam blast, the volume of liquid inside the closed container is increased. This causes a sharp increase in deformation of the circumferential walls of the packaging as evident by the first peaks on the shrinking graphs. The pressure inside decreases as the temperature drops. After that, the dominant load is observed to be generated by the shrinking foil.

The method presented herein makes it possible to determine the influence of many factors on the quality of the shrinking process and its energy consumption. It has been shown that it can be used to select the optimum values of the working medium flow rate. Thus, it makes it possible to optimally select the design of the shrink tunnel itself. In particular it is applicable to individual steam sections. The developed method allows, for example, for the appropriate distribution of steam nozzles in individual sections, the selection of the appropriate shape of these nozzles and the selection of the number of steam sections. All of these factors are closely linked to the reliability of the shrink sleeve labelling process while minimizing production costs.

In industrial practice, a situation should be sought where the clamping force of the label is comparable at each level of the packaging. The force in question is the final force, i.e., after cooling and drying the package. However, it should be emphasized that tests should be carried out for each shape and size of the packaging. This is, of course, cost-effective only in the case of mass production. Out of the results presented in Figure 10, the results from Figure 10a–c,e,g are most advantageous. The differences in the clamping force of the film on three different levels are the smallest here.

## Figures and Tables

**Table 1:** Valve settings for individual steam sections in subsequent measurements.

Measure Series	Valve Settings for Individual Steam Sections											
	1	2	3	4	5	6	7	8	9	10	11	12
1	2.5				2.5				2.5			
2	5				5				5			
3	7				7				7			
4	0				2.5				2.5			
5	0				2.5				0			
6	2				4				7			
7	7				4				2			

## References

[1] Izdebska J., Izdebska J., Thomas S. (2016). Applications of printed materials. Printing on Polymers.

[2] Raheem D. (2013). Application of plastics and paper as food packaging materials? An overview. Emir. J. Food Agric..

[3] Rudnik E., Ebnesajjad S. (2013). Compostable properties and packaging applications. Plastic Films in Food Packaging.

[4] Averous L., Moro L., Dole P., Fringant C. (2000). Properties of thermoplastic blends: STARCH–polycaprolactone. Polymer.

[5] Matzinos P., Tserki V., Kontoyiannis A., Panayiotou C. (2002). Processing and characterization of starch/polycaprolactone products. Polym. Degrad. Stab..

[6] Mo X., Sun X.S., Wang Y. (1999). Effects of molding temperature and pressure on properties of soy protein polymers. J. Appl. Polym. Sci..

[7] Srinivasa P.C., Tharanathan R.N. (2007). Chitin/Chitosan—safe, ecofriendly packaging materials with multiple potential uses. Food Rev. Int..

[8] Tserki V., Matzinos P., Pavlidou E., Vachliotis D., Panayiotou C. (2006). Biodegradable aliphatic polyesters. Part I. Properties and biodegradation of poly(butylene succinate-co-butylene adipate). Polym. Degrad. Stab..

[9] Tserki V., Matzinos P., Pavlidou E., Panayiotou C. (2006). Biodegradable aliphatic polyesters. Part II. Synthesis and characterization of chain extended poly(butylene succinate-co-butylene adipate). Polym. Degrad. Stab..

[10] Wang X.L., Yang K.K., Wang Y.Z. (2003). Properties of starch blends with biodegradable polymers. J. Macromol. Sci. Part C Polym. Rev..

[11] Zhang J., Mungara P., Jane J. (2001). Mechanical and thermal properties of extruded soy protein sheets. Polymer.

[12] Zhang X., Do M.D., Hoobin P., Burgar I. (2006). The phase composition and molecular motions of plasticized wheat gluten-based biodegradable polymer materials studied by solid-state NMR spectroscopy. Polymer.

[13] Szusta J., Karakaş Ö., Tomczyk A. (2018). Experimental investigation of thin films with various overprints used for packaging labels. Theor. Appl. Fract. Mech..

[14] Patterson R., Kandelbauer A., Muller U., Lammer H., Goodman S.H., Dodiuk-Kenig H. (2014). Crosslinked thermoplastics. Handbook of Thermoset Plastics.

[15] Makuuchi K., Cheng S. (2012). Radiation Processing of Polymer Materials and Its Industrial Applications.

[16] Breil J., Ebnesajjad S. (2013). Biaxially oriented films for packaging applications. Plastic Films in Food Packaging.

[17] Kondratov A.P., Varepo I.G., Nagornova I.V., Ermakova I.N. (2015). Transparent Layered Materials Based on Variable Color Polyolefins. Procedia Eng..

[18] Schäfer C.G., Viel B., Hellmann G.P., Rehahn M., Gallei M. (2013). Thermo-cross-linked Elastomeric Opal Films. ACS Appl. Mater. Interfaces.

[19] Timoshenko S., Goodier J.N. (1951). Theory of Elasticity.

[20] Watson B.R., Sharpe W.N. (2008). Bonded electrical resistance strain gages. Springer Handbook of Experimental Solid Mechanics.

